# Heat Shock Protein-70 Levels Are Associated With a State of Oxidative Damage in the Development of Bronchopulmonary Dysplasia

**DOI:** 10.3389/fped.2021.616452

**Published:** 2021-05-26

**Authors:** Chien-Chou Hsiao, Cheng-Han Lee, Rei-Cheng Yang, Jia-Yuh Chen, Tzu-Cheng Su, Yu-Jun Chang, Ching-Yuang Lin, Yi-Giien Tsai

**Affiliations:** ^1^Department of Pediatrics, Changhua Christian Children's Hospital, Changhua, Taiwan; ^2^School of Medicine, Kaohsiung Medical University, Kaohsiung, Taiwan; ^3^School of Medicine, Chung Shan Medical University, Taichung, Taiwan; ^4^Department of Pediatrics, Kaohsiung Medical University Hospital, Kaohsiung, Taiwan; ^5^Department of Pathology, Changhua Christian Hospital, Changhua, Taiwan; ^6^Epidemiology and Biostatistics and Big Data Center, Changhua Christian Hospital, Changhua, Taiwan; ^7^Division of Pediatric Nephrology, Children's Hospital, China Medical University, Taichung, Taiwan

**Keywords:** heat shock protein-70, bronchopulmonary dysplasia, preterm infants, apoptosis, oxidative stress

## Abstract

**Background:** Heat shock protein-70 (Hsp-70) exhibits cytoprotective effects against oxidative stress-induced airway injury. This study aimed to examine Hsp-70 and 8-hydroxy-2′-deoxyguanosine (8-OHdG) from tracheal aspirates (TA) in very low-birth weight (VLBW) preterm infants to predict the development of bronchopulmonary dysplasia (BPD).

**Methods:** This birth cohort study enrolled 109 VLBW preterm infants, including 32 infants who developed BPD. Hsp-70 and 8-OHdG concentrations from TA were measured by immunoassay. The apoptosis of TA epithelial cells obtained on Day 28 after birth was measured using annexin-V staining assay.

**Results:** Hsp-70 and 8-OHdG levels in TA fluid were persistently increased from Day 1 to Day 28 of life in the BPD group. Multiple linear regression analysis demonstrated that BPD was significantly associated with gestational age, respiratory distress syndrome, and TA Hsp-70 and 8-OHdG levels on post-natal Day 28. The TA Hsp-70 level positively correlated with TA 8-OHdG level on the Day 1 (*r* = 0.47) and Day 28 of life (*r* = 0.68). Incubation of recombinant Hsp-70 with primary epithelial cells derived from TA of patients decreased hydrogen peroxide-induced epithelial cell death.

**Conclusions:** Hsp-70 levels are associated with a state of oxidative injury in the development of BPD.

## Introduction

Heat shock proteins (Hsps) constitute a family of molecular chaperones constitutively expressed in virtually all nucleated cells. Hsp-70, a highly inducible chaperone of the Hsps family, plays a key role in protein folding, protein stability, and regulating cellular homeostasis (1, 2). Hsp-70 is enhanced by exposure to various stressful conditions, including heat, oxidative stress, ischemia, and inflammation (1–3). Hsp-70 expression is upregulated by cell injury to keep the cells viable, and the induction of Hsp-70 is a potential therapeutic target against several stressor-induced injuries (4).

Bronchopulmonary dysplasia (BPD) is the most common chronic lung disease of very low-birth weight (VLBW) preterm infants at risk of mortality (5). The prevalence of BPD has remained relatively constant in neonatal intensive care units despite improvements in perinatal care with post-natal surfactant, antenatal corticosteroids, and gentler mechanical ventilation strategies (5, 6). The pathogenesis of BPD is a multifactorial process linked to the arrested development of immature lungs, barotrauma, oxidant injury, inflammation, enlarged airspaces, and mild airway smooth muscle thickening (6–8).

Supplemental oxygen and mechanical ventilation in premature infants may cause oxidative stress damage in the respiratory system by free radicals and limited antioxidants (9, 10). Previous studies have demonstrated the role of elevated levels of oxidative stress in the development of BPD (11, 12). Oxidative stress is strongly related to the cell death process, which may be attributed to the inflammation of premature lung (13). The overexpression of Hsp-70 in alveolar epithelial cells is reported to enhance the resistance to lung injury, and Hsp-70 is gaining much importance due to its key role against apoptosis caused by oxidative stress (14–17). However, the role and mechanism of Hsp-70 in lung oxidative stress injury in BPD is yet to be fully elucidated. So far, there are limited studies indicating that Hsp70 has a cytoprotective function *via* anti-apoptotic and anti-inflammatory effects in murine BPD models (14, 18). 8-hydroxy-2′-deoxyguanosine (8-OHdG) has been shown to be one of the biomarkers of endogenous oxidative DNA damage to predict BPD development (12). We hypothesize that Hsp-70 has cytoprotective effects against oxidative stress injury in the lungs that leads to BPD. This study assessed the time series follow-up between Hsp-70 and 8-OHdG levels from tracheal aspirates (TA) in the development of BPD among VLBW infants. The abovementioned results from previous studies may provide substantial evidence to understand the role of Hsp-70 in premature lung development and promote a comprehensive prevention strategy for BPD.

## Materials and Methods

### Patient Populations

For this prospective cohort study (April 4, 2015 to March 30, 2019), we collected VLBW infants with gestational age < 32 weeks, weight < 1,500 g, and respiratory failure by mechanical ventilation in the neonatal intensive care unit of Changhua Christian Children's Hospital. The patients were initially managed on conventional mechanical ventilators or with high-frequency oscillatory ventilation for those who showed poor response to conventional ventilation. Demographic data, including birth weight (BW), gestational age, gender, Apgar score, Cesarean delivery, requirement of ventilator days, presence of retinopathy of prematurity, prenatal and postnatal steroids, sepsis, patent ductus arteriosus (PDA), respiratory distress syndrome, surfactant therapy, intraventricular hemorrhage, and necrotizing enterocolitis (NEC) were reviewed from chart records of the patients. BPD is defined as the need for respiratory support with an oxygen supplement of more than 21% oxygen and for a high-flow nasal cannula after 36 weeks post-menstrual age (PMA). Using 21% oxygen, continuous positive airway pressure (CPAP) ventilation was not included in the BPD diagnosis (6). This study aimed to compare the relationship between the Hsp-70 and 8-OHdG levels from tracheal aspirates (TA) and the development of BPD among VLBW infants.

The exclusion criteria were (1) congenital abnormalities; (2) anatomic obstructive gastrointestinal disease; (3) congenital heart disease; (4) hereditary metabolic disorder; and (5) early expiry within 7 days. The medical care of the infants was conducted by the same attending physician, and the physicians did not know the Hsp-70 and 8-OHdG values of the patients. The study was approved by the Institutional Review Board (Nos. 130114 and 180201) at Changhua Christian Hospital, and the parents of the infants provided informed consent.

### Neonatal Tracheal Aspirates and Cell Culture and Serum Collection

The TA samples were collected using our previously published protocol in accordance with American Thoracic Society guidelines (12, 19). Tracheal suctioning in intubated infants was performed by the respiratory therapist and nurse as needed to maintain airway patency. TA collection in the patients weaned from the ventilator at Day 28 was performed by the attending physician using direct laryngoscopy, and a passage was made into the trachea with an 8-Fr suction catheter to avoid oropharyngeal contamination. All infants tolerated this procedure well without any obvious desaturations, bradycardia, apnea, or other complications. The TA samples were transported on ice and processed within 30 min in the laboratory and centrifuged at 4°C for 10 min at 300 *g*. The supernatant was collected, divided into aliquots, and stored at −80°C for future use. Primary airway epithelial cell cultures were isolated from TA cell pellets and cultured as previously described (20, 21). Primary airway epithelial cells cultured in 2 mL Dulbecco's Modified Essential Medium with 100 IU/mL penicillin, 100 μg/mL streptomycin, 2 mM L-glutamine, and 0.25 mg/ mL amphotericin B (Gibco, Paisley, UK) in a humidified atmosphere containing 5% CO_2_ at 37°C. Neonatal serum from blood samples (1.0 mL) was obtained within 24 h and at Day 28 following birth.

### Enzyme-Linked Immunosorbent Assay

Total protein concentration was measured in each TA sample by the Bradford assay (Bio-Rad, Richmond, CA) to correct for the dilution according to our previous published protocol (12). The level of Hsp-70 in the serum and TA fluid were examined using ELISA (R&D Systems, Minneapolis, MN). Oxidative stress marker formation of 8-OHdG by oxygen radicals was measured using a highly sensitive 8-OHdG ELISA kit (JalCA, Fukuroi, Shizuoka, Japan). The level of 8-OHdG and Hsp-70 were expressed as “ng/mg” and “pg/mg” of protein, respectively.

### Annexin-V/Propidium Iodide Double Staining and Flow Cytometry

To detect the role of Hsp-70 in H_2_O_2_-induced apoptosis in primary respiratory epithelial cells from TA in BPD, an FITC annexin-V/propidium iodide (PI) Apoptosis Detection Kit I (BD Pharmingen, USA) was used. The TA sample from 10 preterm infants diagnosed with severe BPD at 36 weeks PMA was strictly matched to the TA sample of 10 control subjects. Recombinant human Hsp-70 protein with low endotoxin (Enzo Life Science, Farmingdale, NY) (5 μg/ml) was incubated with primary epithelial cells (1 × 10^5^ cells) from TA and then cultured with H_2_O_2_ (0.5 mM) for 1 h, followed by 8 h of recovery. Cells were stained with PI and FITC annexin-V for 15 min according to the protocols of the manufacturer and then subjected to flow cytometry analysis (FC500, Beckman Coulter, Fullerton, CA) (22).

### Statistical Analysis

All continuous variables were presented as median and interquartile range (IQR), whereas categorical variables were presented as absolute (*n*) and relative (%) frequencies. Laboratory data are presented as mean ± SD. To assess whether clinical background variables differed between preterm infants with and without BPD, the Chi-square test or Fisher's exact test was used for categorical data, and the Mann–Whitney *U*-test was used for continuous data. Within-group comparison of changes from baseline was carried out by means of the two-sided Wilcoxon signed rank test. Groups of data sets were compared using the Kruskal–Wallis test, followed by the Duncan test. The relationship between variables was evaluated using Spearman's rank correlation coefficient. Finally, logistic regression to predict factors associated with BPD was used. The multiple regression models were performed to evaluate the association between BPD and variables adjusted for several potential confounders, such as gestational age, birth weight, BMI, Apgar scores, surfactant use, ventilator days, O_2_ days, RDS, TA 8-OHdG, and TA Hsp-70. A *p* < 0.05 was considered statistically significant.

## Results

### Demographic Characteristics and Clinical Outcomes

In total, 156 preterm infants were screened and 109 VLBW preterm neonates were selected, including 80 who developed respiratory distress syndrome (RDS) and 32 who developed BPD at postmenstrual age 36 weeks (Supplementary Figure 1). As the demographics characteristics show (Table 1), the patients who developed BPD were lighter in weight, were more immature, and had RDS that needed surfactant treatment. Neonates with 1 and 5 min Apgar scores were lower in BPD group. The mechanical ventilation and oxygen therapy periods were significantly longer in the BPD group. The frequency of retinopathy of prematurity and intraventricular hemorrhage were less in the non-BPD group as shown in Table 1 (*P* < 0.05).

**Table 1:** Demographic characteristics and clinical outcomes.

	**Non-BPD**	**BPD**
	**(*n* = 77)**	**(*n* = 32)**
Male gender, n%	42 (55%)	18 (56%)
Gestational age, wk	28.0 (27–31)	27.0 (25–28.5)^*^
Birth weight, g	1,060 (961–1,247)	838.5 (647–1,014)^*^
Cesarean delivery, n%	39 (70.1%)	22 (68.8%)
Antenatal steroid, n%	50 (64.9%)	24 (75%)
Respiratory distress syndrome, *n*%	54 (70.1%)	26 (81.3%)^*^
Surfactant use, *n*%	31 (40.3%)	22 (68.7%)^*^
1-min Apgar score	6 (1–8)	5 (1–7)^*^
5-min Apgar score	8 (2–9)	7 (3–9)^*^
Day 1 blood WBC (10^3^/μl)	14.1 (10.3–17.8)	15.5 (12.2–24.1)
Ventilator days	6 (2–30)	30 (9–57)^*^
O_2_ days	42.0 (24–51)	82 (38–100)^*^
ROP stage III-IV, *n*%	1 (1.3%)	2 (6.2%)^*^
NEC, *n*%	1 (1.3%)	1 (3.1%)
PDA, *n*%	41 (53.2%)	20 (62.5%)
Sepsis, *n*%	5 (6.5%)	5 (15.6%)
Intraventricular hemorrhage grades III-IV, *n*%	2 (2.6%)	3 (9.4%)^*^

*Data are presented as number (percentage) or median (IQR)*.

*Differences between the two groups were assessed by the Fisher exact test for categorical data and the Mann–Whitney U-test for continuous variables*.

* *Mean P < 0.05*.

*NEC, necrotizing enterocolitis; ROP, retinopathy of prematurity; PDA, patent ductus arteriosus; IQR, interquartile range*.

### Increased Hsp-70 and 8-OHdG in TA From VLBW Infants Who Develop BPD

**Figure 1:**
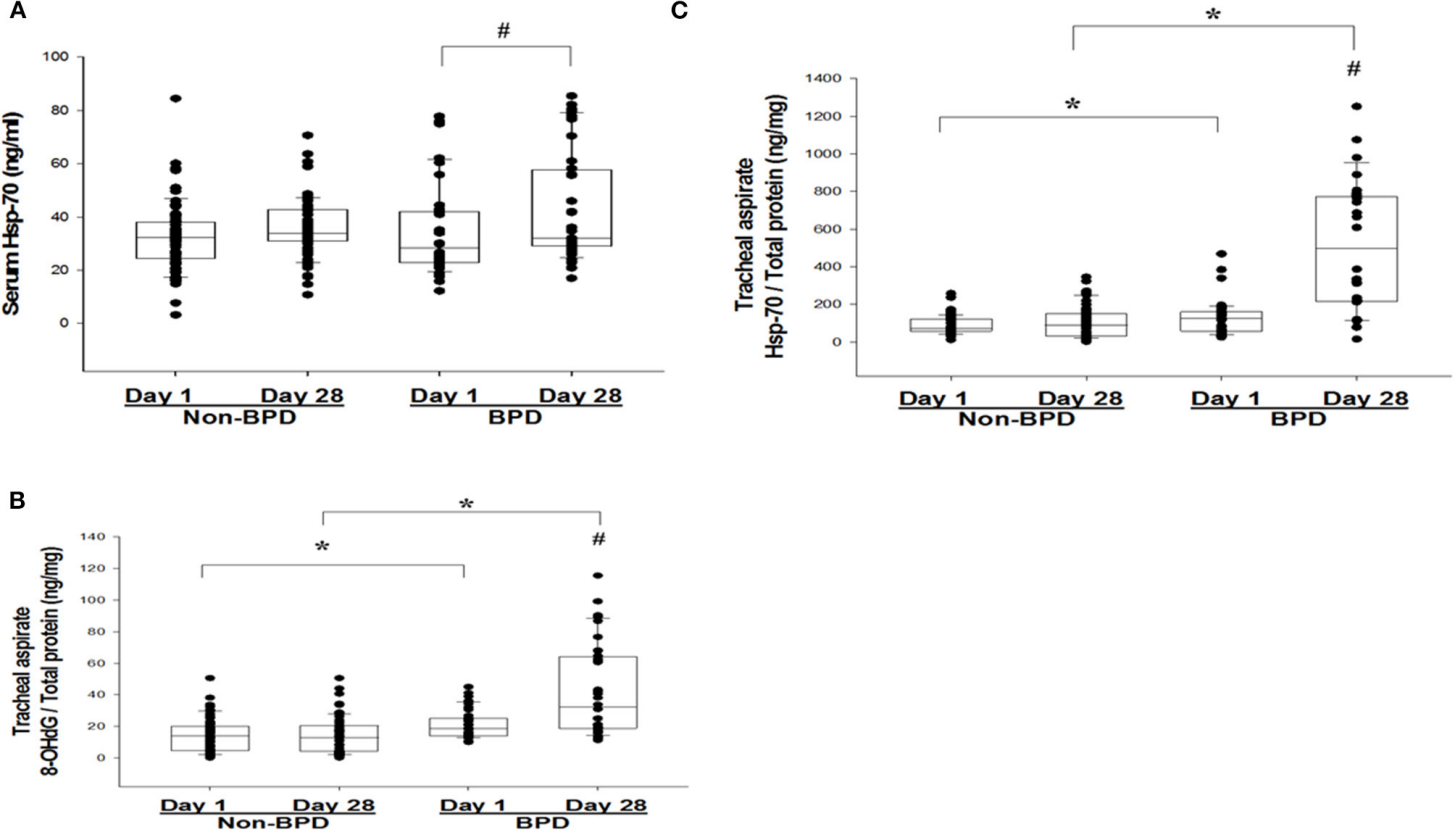
**(A)** Serum heat shock protein-70 (Hsp-70) on post-natal days 1 and 28 from very low–birth weight (VLBW) infants who developed bronchopulmonary dysplasia (BPD). **(B)** 8-hydroxy-2′-deoxyguanosine (8-OHdG) and **(C)** Hsp-70 from tracheal aspirates (TAs) on post-natal days 1 and 28 from VLBW infants, according to the absence or presence of BPD. The box plots show the 25th and 75th percentiles. ^#^*P* < 0.05 between Day 1 and Day 28. *P < 0.05 between the BPD and non-BPD groups.

Serum Hsp-70 was significantly higher on Day 28 than on Day 1 in the BPD group (*P* < 0.05) (Figure 1A). In TA fluid, the oxidative stress marker with 8-OHdG was significantly higher in the BPD group compared with the non-BPD group on Day 1 of life (20.9 ± 8.9 vs. 14.8 ± 10.4 ng/mg, *P* < 0.05; Figure 1B) and on Day 28 (42.0 ± 28.5 vs. 14.1 ± 10.6 ng/mg, *P* < 0.05; Figure 1B). In the same condition, TA Hsp-70 was significantly higher in the BPD group compared with the non-BPD group on Day 1 (126.4 ± 90.7 vs. 87.3 ± 45.0 pg/mg, *P* < 0.05) and on Day 28 (502.1 ± 330.0 vs. 105.2 ± 81.1 ng/mg, *P* < 0.05) of life (Figure 1C). Both Hsp-70 and 8-OHdG in TA fluid were persistently higher on post-natal Day 28 than on Day 1 in the BPD group (*P* < 0.05) (Figures 1B,C).

### TA Hsp-70 and 8-OHdG Are Biomarkers to Predict the Development of BPD

**Figure 2:**
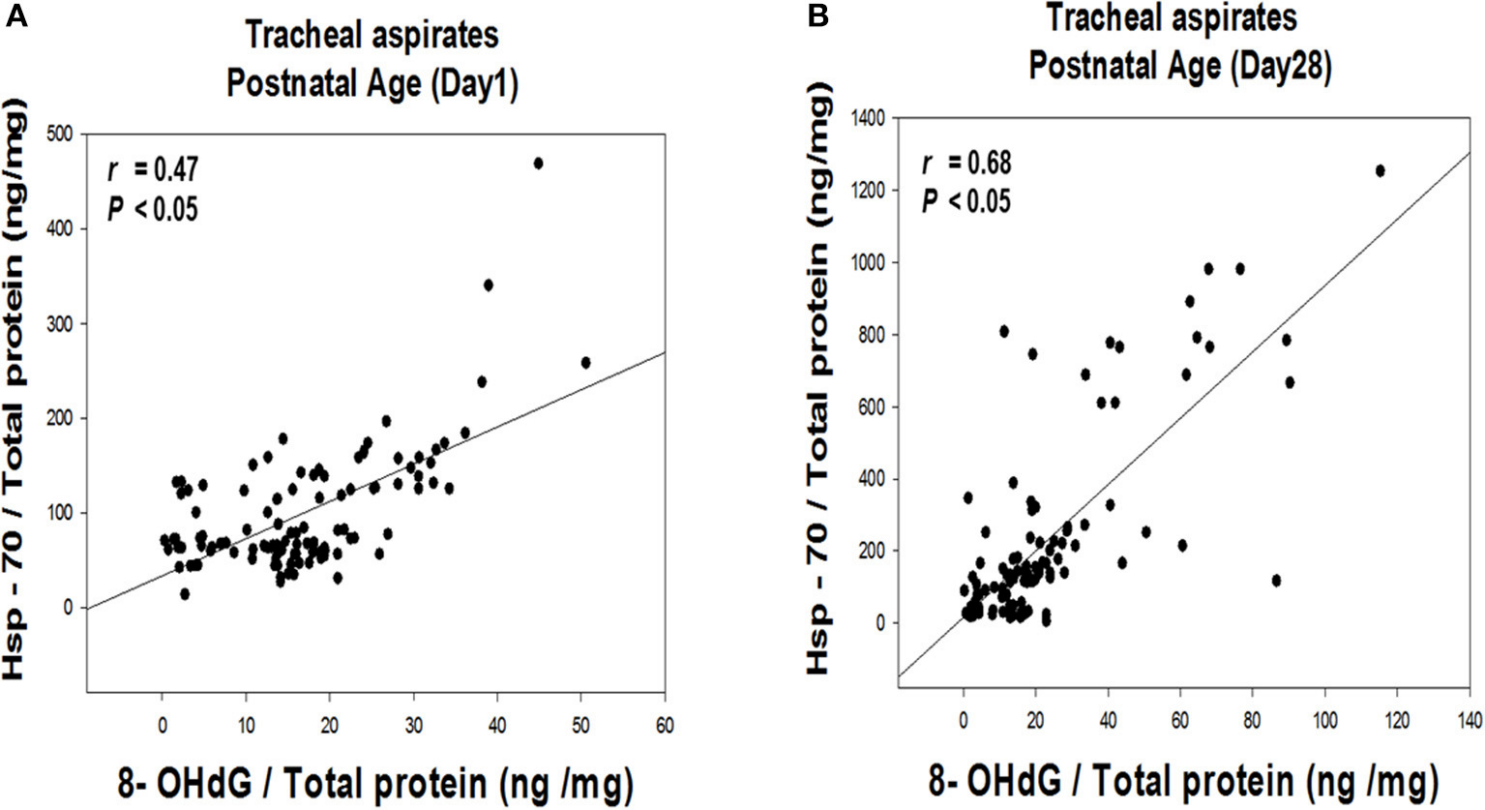
Correlation between TA Hsp-70 and 8-OHdG levels on post-natal Day 1 **(A)** and Day 28 **(B)** of life.

**Figure 3:**
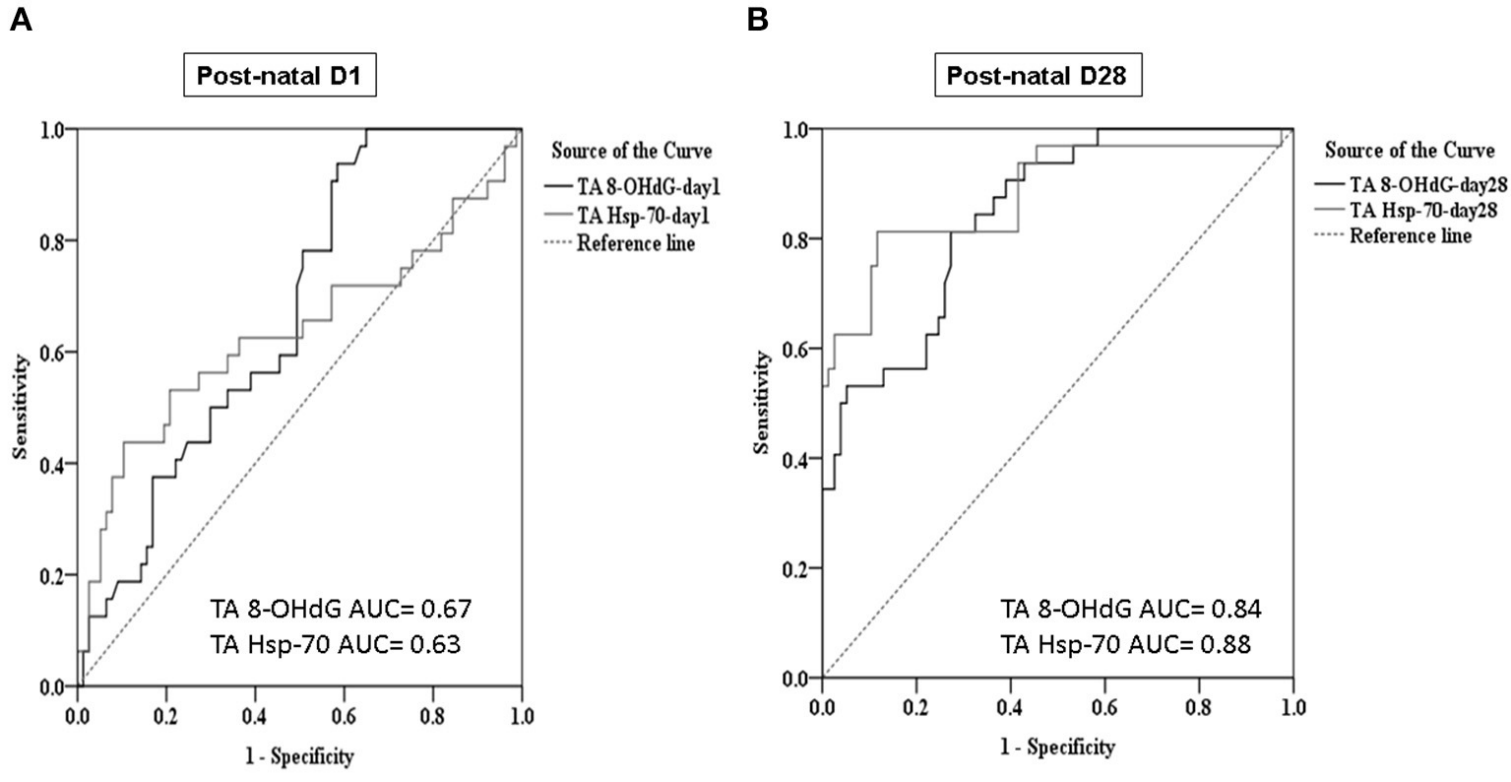
Cutoff values of TA Hsp-70 and 8-OHdG levels discriminate BPD in VLBW preterm infants on post-natal Day 1 **(A)** and Day 28 **(B)** of life.

Multiple linear regression analysis demonstrated TA Hsp-70 and 8-OHdG levels on post-natal Day 28, gestational age, and respiratory distress syndrome were significantly associated with BPD diagnosis (*P* < 0.05) (Table 2). The TA Hsp-70 positively correlated with 8-OHdG levels on post-natal Day 1 (*r* = 0.47, *P* < 0.05) and Day 28 (*r* = 0.68, *P* < 0.05) (Figures 2A,B). Since TA Hsp-70 and 8-OHdG measurements can be useful in diagnosing BPD, we used the receiver–operating characteristic curve to determine the degree of significance for the differential diagnosis. Based on the ROC curves of the predictive effect on BPD, TA 8-OHdG levels on post-natal Day 1 (cutoff, 12.7 ng/mg) showed a sensitivity of 93.8%, a specificity of 41.6%, and an area under curve of 0.67. In the same condition, TA Hsp-70 on post-natal Day 1 (cutoff, 138.8 ng/mg) had 43.8% sensitivity, 89.6% specificity, and 0.63 area under the curve (AUC) (Figure 3A). The cutoff TA 8-OHdG for BPD diagnosis on post-natal Day 28 with high sensitivity (81.3%) and high specificity (72.7%) was 18.3 ng/mg and an AUC of 0.84. The cutoff TA Hsp-70 for BPD diagnosis on post-natal Day 28 with high sensitivity (81.3%) and high specificity (88.3%) was 206.5 ng/mg and an AUC of 0.88 (Figure 3B). In addition, we found the cutoff value of increased TA Hsp-70 level (over 149.1 ng/mg) with 71.9% sensitivity and 93.5% specificity to diagnose BPD patients, indicating a good predictive effect on BPD.

**Table 2:** Regression analysis for the TA Hsp-70 and 8-OHdG levels in patients with bronchopulmonary dysplasia.

	**Univariate analysis (crude)**					**Multiple analysis (adjusted)**				
	**Odds ratio**	**95% C.I**.			***P*-value**	**Odds ratio**	**95% C.I**.			***P*-value**
GA	0.702	0.573	–	0.860	0.001	0.707	0.507	–	0.986	0.041
BW	0.995	0.992	–	0.997	<0.001					
Apgar score (1-min)	0.752	0.595	–	0.949	0.017					
Apgar score (5-min)	0.663	0.493	–	0.892	0.007					
Surfactant use (Yes vs. No)	7.778	2.736	–	22.109	<0.001	54.607	1.609	–	1853.712	0.026
Ventilator days	1.050	1.027	–	1.074	<0.001					
O_2_ days	1.071	1.044	–	1.100	<0.001					
RDS (Yes vs. No)	4.685	1.734	–	12.657	0.002					
ROP (Yes vs. No)	5.088	2.103	–	12.309	<0.001					
TA 8-OHdG-day 1	1.062	1.017	–	1.108	0.006					
TA Hsp-70-day 1	1.010	1.002	–	1.018	0.011					
TA 8-OHdG-day 28	1.093	1.050	–	1.137	<0.001	1.099	1.025	–	1.179	0.008
TA Hsp-70-day 28	1.012	1.006	–	1.018	<0.001	1.011	1.003	–	1.019	0.005
Δ TA 8-OHdG	1.068	1.033	–	1.104	<0.001					
Δ TA Hsp-70	1.011	1.006	–	1.016	<0.001					

*Δ = day 28–day 1*.

*BW, birth weight; GA, gestational age; RDS, respiratory distress syndrome; TA, tracheal aspirates*.

### Recombinant Hsp-70 Attenuates Hydrogen Peroxide-Induced TA Epithelial Cell Death in BPD Patients

**Figure 4:**
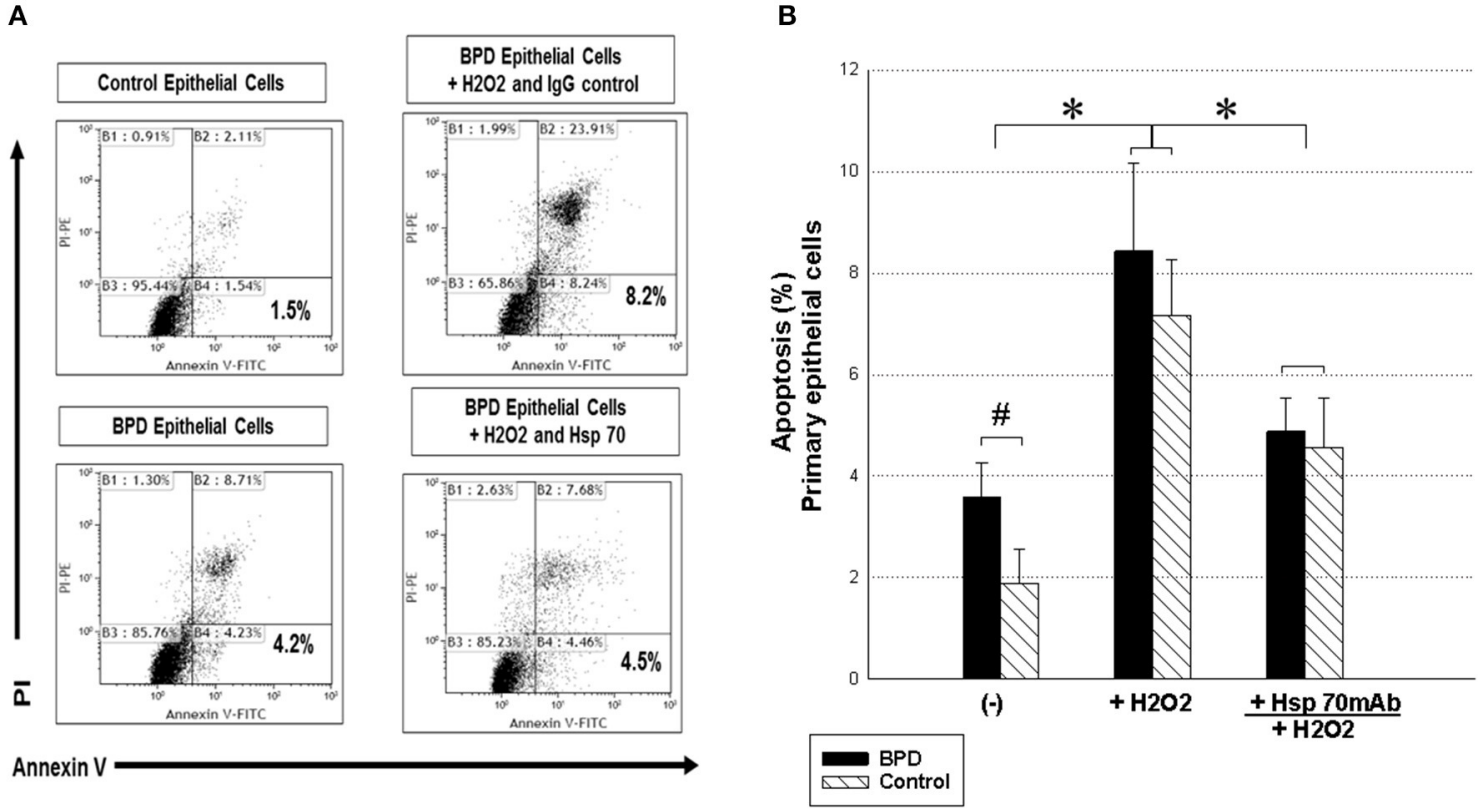
Recombinant human Hsp-70 decreases H_2_O_2_-induced airway epithelial cell apoptosis from TA in the controls and patients with BPD. **(A)** To detect the role of Hsp-70 in early apoptotic cells with annexin-V-positive but PI-negative respiratory epithelial cells from TA after exposure to hydrogen peroxide, a FITC annexin-V/propidium iodide Apoptosis Detection Kit I (BD Pharmingen, USA) was used. Recombinant human Hsp-70 (5 μg/ml) was incubated with primary epithelial cells (1 × 10^5^ cells) from the TA of patients with BPD and of control subjects and then cultured with H_2_O_2_ (0.5 mM) for 1 h followed by 8 h of recovery. Representative figures are shown. **(B)** Statistical data of the experiments with 10 paired samples. The Kruskal–Wallis test was used to determine significant differences. *p < 0.05 after treatment and ^#^*p* < 0.05 compared to the control group.

Heat shock proteins are gaining much importance due to their potential to be key determinants of cell survival and apoptosis. We tested the hypotheses that respiratory epithelial cells exposed to recombinant human Hsp-70 would be less susceptible to lung injury with oxidative stress. To clarify the anti-apoptosis mechanisms involved in the Hsp-70, we examined the apoptosis of respiratory epithelial cells *via* the PI/annexin-V assay from TA of patients with BPD. An increased apoptosis rate with annexin-V expression from the TA primary epithelial cells was found in patients with BPD (*P* < 0.05) (Figures 4A,B). In the analysis of the hydrogen peroxide-mediated cells with annexin-V expression, the percentage of apoptosis of recombinant Hsp-70 pretreated primary epithelial cells from the TA of patients with BPD was decreased compared with the IgG control group in patients with BPD (4.8 ± 0.6 vs. 8.4 ± 1.7%, *p* < 0.05; Figure 4A). Experiments were performed with 10 paired samples, and the statistical data are shown in Figure 4B.

## Discussion

Mechanisms affecting the development of BPD may be due to imbalanced pro-inflammatory and anti-inflammatory mechanisms in the premature respiratory tract system (6, 7). Both prolonged hyperoxia exposure and lung-stretching by ventilator support will lead to the production of highly reactive oxygen radicals and pro-inflammatory cytokines, which may cause acute lung injury and chronic pulmonary remodeling in BPD (23). Hsp-70, which has shown cytoprotective effects, is able to migrate to the circulation from cells under oxidative stress, the DNA damage response, and many other stress conditions (1–3). Several studies demonstrated that changes in Hsp-70 are associated with chronic obstructive pulmonary disease and various inflammatory diseases (15, 24, 25). In our results, VLBW preterm infants who developed BPD had significantly higher serum and TA Hsp-70 concentrations compared with infants who did not develop BPD. We further demonstrated that BPD diagnosis was significantly associated with TA Hsp-70 and 8-OHdG levels on post-natal Day 28 by multiple linear regression analysis. Lower Apgar scores, higher incidence of RDS, and surfactant use are indicative of higher perinatal stress, which may cause Hsp-70 production. In addition, persistent ventilator injury and oxygen toxicity may enhance Hsp-70 expression. Upregulating Hsp-70, which has been closely associated with the post-translational repair process, may protect against oxidative injury in BPD. The study provides further evidence of a link between Hsp-70 and 8-OHdG levels in the development of BPD, highlighting the importance of targeting molecular chaperone and crucial cytoprotective capacity to prevent oxidative distress and DNA damage in the respiratory tract.

Oxidative stress increases the levels of reactive oxygen species and reduces membrane potential in respiratory epithelial cells that may trigger apoptosis. Oxidative stress with persistent DNA damage and apoptosis in the respiratory system may be an important mechanism in the pathogenesis of BPD (18). Hsp-70 represents key mediators in the chaperone network with diverse functions in stabilizing unfolded proteins, anti-inflammatory properties, and promoting growth and survival of the respiratory cells (4, 26, 27). The induction of Hsp-70 is a potential therapeutic target against several stressor-induced lung injuries in BPD. Some reports demonstrated that increased Hsp-70 expression in lungs protects alveolar epithelial cells against apoptosis and antagonizes the hyperoxia injury (14, 17, 18). Increasing evidence suggests that overexpression of Hsp-70 alleviates cell apoptosis *via* the mitogen-activated protein kinase (MAPK) signaling pathway, and exogenous extracellular Hsp-70 can prevent caspase-3 activation to inhibit cell apoptosis *via* Toll-like receptors and TRIF-nuclear factor kappa B pathway (14, 27–29). The results indicate that administration of recombinant human Hsp-70 with primary epithelial cells derived from TA significantly inhibited decreased respiratory epithelial cell apoptosis by hydrogen peroxide-induced oxidative stress; therefore, targeting Hsp-70 is possibly a good strategy to develop anti-oxidative therapeutics in patients with BPD.

Gestational age, birth weight, and male gender are known risk factors for the development of BPD. We demonstrated that male VLBW premature infants had a high percentage of RDS (66.1 vs. 46.8%, *P* < 0.05). However, we did not find male preterm infants at risk for BPD development (*P* > 0.05) (Supplementary Table 1). In animal models, Hsp-70 has been reported to be positively regulated by estrogen (30). In this study, there was no sex difference on TA Hsp-70 levels between postnatal Days 1 and 28 (Supplementary Table 2). In contrast, we found that male premature infants showed a significantly increased Hsp-70 compared with female subjects, regardless of BPD diagnosis (147.6 ± 262.3 vs. 90.3 ± 235.8 pg/mg, *P* < 0.05). In this non-BPD premature infant cohort, we found a trend of increase in TA Hsp-70 from post-natal Day 1 to Day 28, but this result did not show a significant difference (Figure 1C).

This study had some limitations. First, this is a single-center cohort study with only 32 VLBW preterm infants who developed BPD. Second, Day 1 TA and/or serum Hsp-70 seems be a more favorable biomarker for BPD in clinical practice, especially as some non-BPD premature infants may be weaned from the ventilator before post-natal Day 28.

In conclusion, the present study demonstrates that increased TA 8-OHdG and Hsp-70 levels are associated with a higher prevalence of BPD. Hsp-70 has potential utility as a biomarker for diverse physiological and pathological conditions in BPD. Hsp-70 can protect apoptosis against the adverse effects of an oxidative stress environment. Therefore, Hsp-70 has promising potency as a therapeutic target in managing BPD. However, more experimental and human studies are warranted to prove its benefit before it can be used in preterm infants. Further understanding regarding the role of Hsp-70 in BPD pathogenesis may disclose new possibilities to modulate Hsp-70 in the future.

## Data Availability Statement

The raw data supporting the conclusions of this article will be made available by the authors, without undue reservation.

## Ethics Statement

The studies involving human participants were reviewed and approved by Institutional Review Board (No:130114 and 180201) at Changhua Christian Hospital. Written informed consent to participate in this study was provided by the participants' legal guardian/next of kin. Written informed consent was obtained from the individual(s), and minor(s)' legal guardian/next of kin, for the publication of any potentially identifiable images or data included in this article.

## Author Contributions

C-HL, R-CY, J-YC, C-CH, T-CS, Y-JC, C-YL, and Y-GT conceptualized and designed the study, performed the experiments, and drafted the manuscript. All authors read and approved the final manuscript.

## Conflict of Interest

The authors declare that the research was conducted in the absence of any commercial or financial relationships that could be construed as a potential conflict of interest.
