# Supplementary material for: Heat Shock Protein-70 Levels Are Associated With a State of Oxidative Damage in the Development of Bronchopulmonary Dysplasia
Source: Front Pediatr. 2021 May 26;9:616452. doi: 10.3389/fped.2021.616452 (PMC8187579; doi:10.3389/fped.2021.616452)
Supplement: Supplementary file 1 [file Table_1.DOCX]

Supplementary Figure 1*.* Enrollment process.


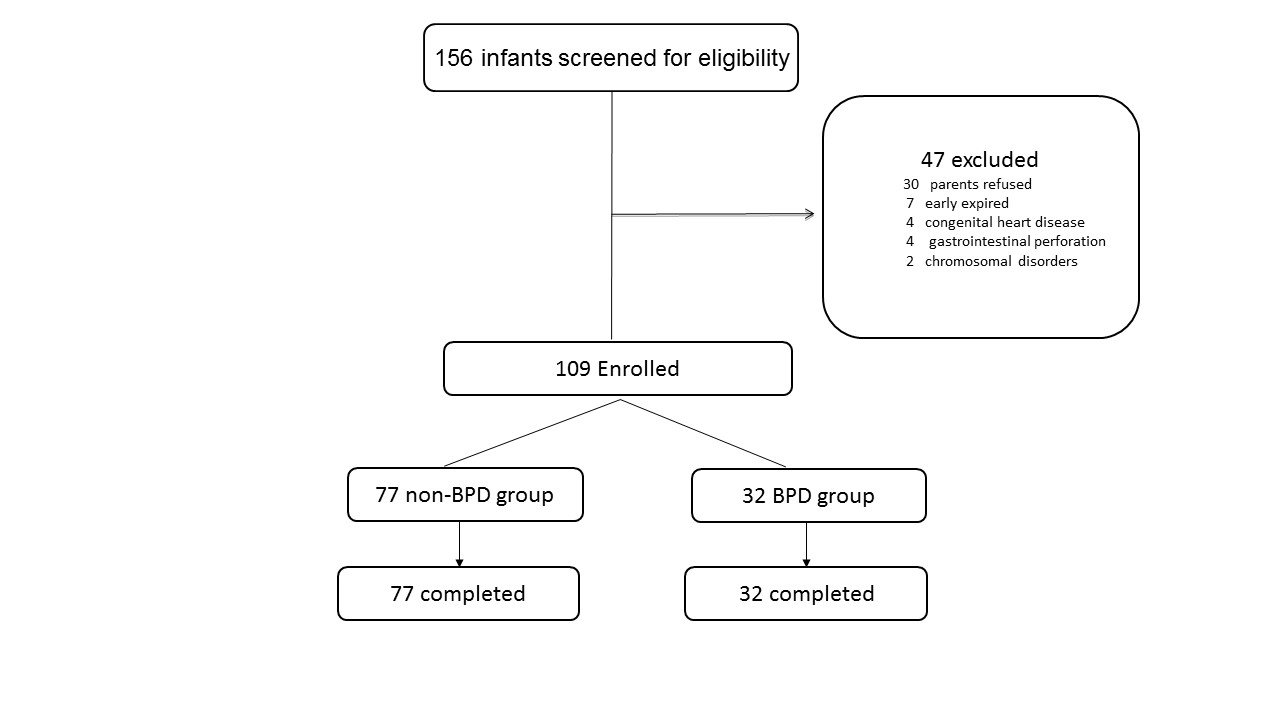


Supplementary Table 1. Gender differences in clinical outcomes.

Supplementary Table 2. Gender differences in TA Hsp-70 and 8-OHdG levels.
